# Does company compliance with RS-17 influence the characterization of a casual nexus in expert testimony?

**DOI:** 10.1590/bjpt-rbf.2014.0071

**Published:** 2015

**Authors:** Manuela Ribeiro, Bruno Guimarães, Breno Sampaio

**Affiliations:** 1 Fisioterapeuta, Recife, PE, Brazil; 2 Programa de Pós-Graduação em Design, Universidade Federal de Pernambuco (UFPE), Recife, PE, Brazil; 3 Programa de Pós-Graduação em Economia, UFPE, Recife, PE, Brazil

**Keywords:** legislation, occupational physical therapy, cumulative trauma disorders, expert testimony, causal nexus, physical therapy

## Abstract

**Objective:**

To examine whether company compliance with RS-17 influences the characterization of the casual nexus in physical therapists' expert reports of cumulative trauma disorders in the labor court of Pernambuco, Brazil.

**Method:**

The sample was composed of seven physical therapists who provided expert testimony regarding cumulative trauma disorder cases in the labor court of Pernambuco, Brazil. Data collection was performed across two stages. In the first stage, the experts answered a sociodemographic survey and requested the identification numbers of recent cases where expert testimony was provided to characterize the causal nexus. In the second stage, the researchers went to the labor court to collect expert testimony data. These experts indicated that of 75 total cases, 31% (N=23) of the companies fulfilled RS-17, whereas 69% (N=52) did not comply with the law.

**Results:**

Among the organizations that complied with legislation, 30% of the analyzed expert testimonies showed a positive causal nexus. However, of the companies that did not comply with RS-17, 71% of the expert testimonies revealed a causal nexus. These results indicate that the breach of the law increases the probability that a causal nexus will be determined by 54.8%.

**Conclusion:**

The results showed that failure to comply with RS-17 significantly increases the probability that a causal nexus will be determined in physical therapists' expert testimony of cumulative trauma disorders.

## Introduction

The Brazilian *Ministério do Trabalho e Emprego* (MTE) approved regulatory standards (RSs) that specify the consolidation of labor law (CLL) topics and those governing workplaces^1^. These standards are intended to establish rights for employers and employees as well as aim to provide a healthy working environment, prevent health risks, and promote employee safety^2^.

The RSs related to occupational health and safety are obligatory for companies as well as legislative and judicial agencies with employees covered by the CLL^1^. Thus, compliance with health, hygiene, and safety standards is a public matter for both employers and employees for any work activity^3^.

In this context, compliance with these standards can be established via forensic examinations, and these investigations can reveal a company's intention in a preventive context. Within the context of repetitive strain injury/work-related musculoskeletal disorders (RSI/WMSDs; RS no. 17 [RS-17]), which addresses ergonomics, these standards are essential for the physical therapists performing the forensic examination work^4^. RS-17 aims to establish parameters that adapt working conditions to employees' psychophysiological characteristics to provide maximum comfort and safety as well as efficient performance^4^. Therefore, this standard establishes minimum parameters for companies to adopt regarding lifting, transporting, and unloading materials, furniture, and equipment as well as those regarding workplace environment conditions and the organization of the work itself^4^.

Non-compliance with RS-17, in addition to leading to fines imposed by the MTE, has lead to accidents and the development of RSI/WMSD. In this regard, employees who develop RSI/WMSD can appeal to the labor court given their rights regarding damage to their productive lives^5^. When doubt exists in these lawsuits with regard to the presence or cause of an employee-claimed illness or functional impairment, Veronesi^6^ states that the judge is assisted by trained professionals who analyze the likelihood of causality via forensic examination to resolve the dispute.

These examinations occur when an employee (or former employee) files a lawsuit for damages against the company for which he or she worked (i.e., when an individual claims that his or her injury was caused by a work-related activity)^7^. Therefore, this type of forensic examination aims to clarify whether a causal relationship exists between the conditions and tasks performed at work and the injury or functional change. In other words, the influence of work activity characteristics on physical functional capacity and the ability to work must be taken into account^8^.

In this sense, establishing a causal nexus (i.e. the relationship between the cause of the injury and the effect of the injury on performance) supports the link between the conduct of the agent and the result produced by it (i.e., the relationship between the injury and the work)^9^. In cases where employees allege that they acquired RSI/WMSD, these examinations aim to establish a causal nexus (i.e., whether the claimant's injury is related to the activities performed)^6^.

In the past, only doctors performed these examinations but recently, however, examinations by a number of physical therapists who have worked in this area has increased because of their knowledge regarding RSI/WMSD, kinesiology, pathology, biomechanics, and ergonomics. Furthermore, under the rules of the *Conselho Federal de Fisioterapia e Terapia Ocupacional* (COFFITO), physical therapists may legally evaluate functional capacity and determine causation^10^^-^ 12.

To establish causality, physical therapists analyze factors related to the nature of exposure, biomechanical or postural risk, total exposure time, physiomorphopathological characteristics of the injury, the preventive methods adopted by the company to minimize or avoid occupational injury or disease, and epidemiological evidence of the injury or disease in the analyzed sector^6^^,^ 13. With regard to preventive methods, companies must comply with legislation such as the RSs at a minimum.

Based on the above conditions, the current study analyzes whether companies adoption of RS-17 affects the establishment of causality in physical therapists' expert reports of musculoskeletal disorders in the labor courts of Pernambuco to assist these experts in resolving areas of doubt and to increase physical therapist activity in this area.

## Method

This cross-sectional, descriptive, and quantitative study was conducted between November 2013 and January 2014. The Ethics Committee on Human Research at *Hospital Agamenon Magalhães*, Recife, Pernambuco, Brazil, CAAE 19900113.2.000.5197 approved data collection based on research standards, Resolution 466/12.

The sample consisted of physical therapists who perform employee forensic examinations for the labor court of Pernambuco and who work at the labor court of the metropolitan region of Recife, Pernambuco, Brazil. The inclusion criteria defined for the forensic examiners were (a) employment in the labor courts of Pernambuco, Brazil and (b) the completion of five or more employee forensic examinations. The exclusion criterion was refusing to sign the informed consent document. All participants provided informed consent.

Data collection was performed across two stages. In the first stage, participants completed an expert identification form to collect sociodemographic data. This questionnaire asked participants to provide the identification numbers of recent cases (minimum = 5; maximum = 20) in which the expert performed an examination to establish a causal relationship.

In the second stage of the research, the data regarding the forensic examinations performed by the physical therapists for the labor courts were collected. An analysis of the examinations for the indicated cases was performed using expert script analysis (Appendix 1) to facilitate both the collection of information necessary for the study in the most precise and targeted way possible and to better estimate and interpret the econometric model parameters.

An important question addressed in the script concerned compliance with RS-17. Each forensic examination analyzed in this study contained a clear indication that the expert noted that the company did not comply with RS-17. Therefore, knowing the process used by the experts to determine noncompliance with the standard was important. Field research with the experts revealed that a lack of conformity with any RS-17 item was justification for declaring the company as being noncompliant. (The relationship found in the following sections refers to the average effect of noncompliance with the standard upon the establishment of causality.)

### Empirical strategy

As described above, the primary objective of this study was to estimate the effect of compliance with RS-17 on the establishment of a causal nexus in forensic examinations. In this sense, the specified model to estimate the relationship is given by

NEXUS*_i_* = α + ρ.RS-17*_i_* + ε*_i_* (1)

where NEXUS*_i_* is a dummy (binary) variable equal to 1 when causality is established for individual *i* but 0 otherwise; RS-17 is a dummy variable equal to 1 for companies that did not comply with RS-17 and 0 for those that complied; and ε*_i_* is a random term. The parameter of interest is ρ, which represents the difference in the probability of establishing a causal nexus between companies that failed to comply with RS-17 and those that did comply:

ρ* =* E[NEXUS*_i_* | RS-17*_i_* = 0] - E[NEXUS*_i_* | RS-17*_i_* = 1] (2)

However, correct identification of the parameter of interest via Equation 1 require that non-compliance with RS-17 be distributed randomly among firms, and this scenario was clearly not the case^14^. In other words, the company decided whether to comply or not comply with RS-17, and this decision was an endogenous process of the entity (i.e., the entity's choice). Therefore, if companies that were more likely to not comply with RS-17 had different characteristics from those that did comply, then the mean difference calculated above not only represented the effect that noncompliance with RS-17 had on the probability of causality but also the effect of the different unobservable characteristics on this probability^15^.

To minimize the bias noted above, one approach commonly used in the literature is the inclusion of a vector of observable characteristics (**X** *_i_*) in Equation 1. In this case, the least squares multiple regression model would be

### NEXUS*_i_* = α + ρ.RS-17*_i_* + **X** *_i_*`β + ε*_i_* (3)

where ρ represented the difference in the probability of establishing a causal nexus between the companies that did or did not comply with RS-17 in which any difference between the characteristics included in **X*_i_*** was already discounted. In theory, this procedure would lead to an unbiased estimation of the parameter of interest (i.e. the effect of lack of compliance with RS-17) when all of the differences that existed between the companies resulted from differences in X, which is rarely the case.

In seeking to measure the coefficient of interest with the lowest possible bias, we used the propensity score matching (PSM). The aim of PSM is to achieve a better match between observable variables to improve the measure of the desired effect^16^ and to relax linearity assumptions. Rosebaum and Rubin^17^ developed PSM to solve the problem of multidimensionality in a sample's observable matching characteristics. This method can be deployed from a single control variable, the propensity score (*P*[*X*]), defined as the conditional probability of an individual/company to be treated (a), given their observable characteristics^17^, i.e.,

P(X) = Pr(RS-17 = 1 | *X*) (4)

Thus, the average treatment effect on those being treated (ATT), assuming the existence of observable characteristics X (in which conditional units have the same probability of being chosen for the treatment or control groups, i.e., y^RS-17=1^ and y^RS-17=0^ are independent of RS-17 | X), is determined via

ATT = E{E[y^RS-17 = 1^|RS-17 = 1,P(X)]- E[y^RS-17 = 0^|RS-17 = 1,P(X)]|RS-17 = 1} (5)

As Rosebaum and Rubin^17^ stated, the application of the propensity score enables one to adjust the bias that exists between the control and treatment groups. To do so, however, two assumptions must be met:

1) Observable characteristics must be balanced; sample selection requires participation in the program to be independent of the results.
2) Common support must exist, i.e., 0 < *P*(*X*) < 1, so that a treatment group is present for each corresponding control group^16^.

In general, the propensity score, P(X), is not known; therefore, it must be estimated. Using samples from the control and treatment groups, logistic (logit) or probabilistic (probit) regression models are calculated to estimate the probability of individual participation in the treatment group given their observable characteristics.

After estimating the propensity score, subgroups are obtained in the control group with probabilities similar to those of individuals in the treatment group in which the mean differences for each variable are tested between the treated and untreated groups for each score block. If a between-group difference is identified, then a more parsimonious model must be specified for the propensity score estimation. Otherwise, the ATT is calculated when the tests of each variable within each interval indicate that the mean does not significantly differ (i.e., the sample is balanced). Of the various matching methods discussed in the literature, the authors decided to use stratification matching in this study^18^.

## Results

Seven forensic examiner physical therapists participated in the study: two (29%) were male and five (71%) were female, and each had completed a training course on forensic examination. A total of 14% were under 25 years old; 43% were 25 to 30 years old, and 43% were over 30 years old. With regard to length of time since graduation, 14% were between 1 and 3 years since graduation; 28% were between 3 and 5 years since graduation; and 58% had worked more than 5 years since graduation. A total of 14% had worked as an expert for less than a year; 28% had worked as an expert for 3 to 5 years; and 58% had worked as an expert for more than 5 years. Finally, 71% worked at the Recife labor courts; 43% worked at the Olinda labor courts; 14% worked at the Jaboatão dos Guararapes labor courts; and 28% worked at the labor courts in the interior of Pernambuco, Brazil.

The experts reported 75 cases from various labor courts, of which 31% (N=23) complied with RS-17, while 69% (N=52) did not. Table 1 presents the descriptive statistics for companies that did or did not comply with RS-17. First, note that 30% of the forensic examinations of companies that complied with RS-17 determined a causal nexus, whereas 71% of the forensic examinations of non-compliant companies determined a causal nexus.

**Table 1:** Descriptive statistics of the average values of the variables for those companies that complied with RS-17 & those that did not.

Variable	Complied with RS-17	Did not comply with RS-17
Positive nexus	0.30	0.71
	(0.47)	(0.46)
Expert assistant	0.48	0.42
	(0.51)	(0.50)
Complementary exam	0.70	0.75
	(0.47)	(0.44)
Change in exams	0.65	0.73
	(0.49)	(0.45)
Work dismissal	0.96	0.81
	(0.21)	(0.40)
CAT issue	0.26	0.23
	(0.45)	(0.43)
INSS	0.61	0.44
	(0.50)	(0.50)
Physical therapy treatment	0.65	0.50
	(0.49)	(0.50)
Extra labor activity	0.22	0.42
	(0.42)	(0.50)
Inability listed on occupational health certificate	0.13	0.15
	(0.34)	(0.36)
N. Obs.	23	52

The standard deviation for each mean is presented in parentheses.

The results estimated via the least squares method are shown in Table 2. Column 1 shows the correlation between failure to comply with RS-17 and the establishment of a causal nexus (see Equation 1). A positive result indicates a positive correlation between non-compliance with the standard and the determination of a causal nexus. However, this result might be due to a characteristic not considered in the analysis (e.g., whether forensic examinations with regard to RS-17 non-compliance also show higher rates of work absenteeism according to the *Instituto Nacional de Seguridade Social*-INSS, Brazil).

**Table 2:** Estimates of the parameters for each variable included in the model via the ordinary least squares method.

	1	2	3	4	5	6	7	8	9
Noncompliance of NR-17	0.407^***^	0.404^***^	0.404^***^	0.395^***^	0.387^***^	0.372^***^	0.376^***^	0.350^***^	0.350^***^
	(0.116)	(0.118)	(0.119)	(0.123)	(0.118)	(0.119)	(0.109)	(0.118)	(0.067)
Presence of expert assistant		–0.054	–0.063	–0.055	–0.13	–0.137	–0.119	–0.122	–0.122
		(0.110)	(0.115)	(0.123)	(0.116)	(0.113)	(0.113)	(0.116)	(0.079)
Complementary exam			–0.234	–0.239	–0.280^*^	–0.244	–0.243	–0.261	–0.261^**^
			(0.181)	(0.183)	(0.161)	(0.168)	(0.169)	(0.162)	(0.124)
Change in complementary exams				–0.165	0.23	0.203	0.157	0.163	0.163
				(0.185)	(0.158)	(0.158)	(0.174)	(0.174)	(0.153)
Work dismissal				–0.06	–0.121	–0.038	–0.098	–0.158	–0.158
				(0.145)	(0.14)	(0.152)	(0.156)	(0.192)	(0.189)
CAT issue					0.272^**^	0.351^**^	0.301^**^	0.303^**^	0.303^**^
					(0.13)	(0.152)	(0.15)	(0.149)	(0.121)
Dismissal by INSS						–0.181	–0.12	–0.102	–0.102
						(0.141)	(0.141)	(0.141)	(0.16)
Physical therapy treatment							0.05	0.069	0.069
							(0.123)	(0.13)	(0.175)
Inability listed on occupational health certificate							0.332^**^	0.342^**^	0.342^***^
							(0.149)	(0.147)	(0.133)
Extra labor activity								0.114	0.114
								(0.131)	(0.171)
Fixed effect of expert	NO	NO	NO	NO	NO	NO	NO	NO	YES
Constant	0.304^***^	0.330^***^	0.394^***^	0.447^***^	0.456^***^	0.462^***^	0.440^***^	0.457^***^	0.457^***^
	(0.097)	(0.109)	(0.141)	(0.17)	(0.16)	(0.164)	(0.159)	(0.165)	(0.118)
Obs.	75	75	75	75	75	75	75	75	75
R^2^	0.145	0.148	0.164	0.166	0.213	0.236	0.291	0.301	0.301

The standard deviation for each statistic are presented in parentheses. The significance of each estimated parameter is denoted by (*),where

*** represents a significance of 1%,

** represents a significance of 5%, and

* represents a significance of 10%.

To account for other important factors when determining causation, various expert testimony characteristics (e.g., the presence of an expert assistant, the presence of a complementary examination) were added to Equation 1. Estimates for these models are shown in columns 2 through 8 in Table 2. The results supported the hypothesis that a positive relationship existed between non-compliance with the standard and the establishment of causality. Finally, fixed expert effects were added in column 9. The primary purpose of this estimation was to isolate any expert characteristic that was correlated with RS-17 non-compliance and the establishment of causality that might bias the equation shown in column 8^14^. The quantitative result was the same, and reinforced the results described above.

As the Methods section described, we decided to use observable characteristics matching because this protocol was the most robust identification procedure. Therefore, estimating the probability of non-compliance with RS-17 became necessary, taking into account the observable characteristics of the forensic examinations, i.e., the propensity score, P(X), via the logit or probit regression model.

The results of the logit model, whichwas more commonly recommended in the literature, are presented in Table 3. Note that the companies that did not comply with RS-17 had higher rates of absenteeism and non-work related activity performance among the employees examined.

**Table 3:** Estimation of the pairing procedure logit model to calculate Propensity Score Matching (PSM).

	Estimated Parameter	Standard Deviation
Expert assistant	–0.330	0.61
Complementary exam	–0.612	1.01
Change in exams	1.227	1.08
Work dismissal	–1.996^*^	1.22
CAT issue	0.518	0.71
INSS	–0.099	0.65
Physical therapy treatment	–0.524	0.65
Extra labor activity	1.164^*^	0.62
Inability listed on occupational health certificate	0.180	0.81
Constant	2.182^**^	1.16
R2	0.335	
Obs.	75	

Estimating performed via the maximum probability for logit model aiming at the calculation of the propensity score Matching. The column 2 shows the value of the estimated parameter and column 3 the standard deviation of this estimation. INSS: National Institute of Social Security; CAT: Communication of Injury; R2: indicates how well the data fit the statistical model. It is value increase when additional independent variables are included in the model; Obs: Number of observations. The significance of each estimated parameter is detonated by (*), where

** represents the significance of 5% and

* represents the significance of 10%.

After calculating the probability of RS-17 non-compliance using the probability model presented in Table 3, the examinations were matched with regard to observable characteristics. That is, the examinations were grouped based on the probabilities of RS-17 non-compliance given the observable characteristics; thus, causal nexus comparisons were made using as many uniform units as possible.

Table 4 presents the final PSM result, comparing homogeneous examinations to more accurately calculate the difference in the probability of having determined a causal nexus due to RS-17 non-compliance. The final result indicates that non-compliance with the standard was associated with a 54.8% increase in the probability of determining a causal nexus.

**Table 4:** Causal effect estimation of compliance with RS-17 via the Propensity Score Method (PSM) model. The parameter of interest is the paired difference between the probability of a causal nexus among companies that failed to comply with RS-17 (treatment) and the probability of a causal nexus among companies that complied (control).

	Sample	Treatment	Control	Paired Difference	Standard Deviation
Nexus	Unmatched	0.712	0.304	0.407^***^	0.116
	Matched	0.712	0.163	0.548^***^	0.178

*** represents the significance of 1%.

## Discussion

The aim of RSs is to guide organizations with regard to the care and diligence that they must adopt toward their employees to prevent occupational accidents and work-related injuries or diseases in Brazil. Compliance with RSs is mandatory for employers.

The INSS Workplace Accidents Statistical Yearbook presents the current landscape of employee safety and health in Brazil. In 2011, 711,164 accidents occurred; of these accidents, 15,083 were due to work-related injuries/diseases. A total of 2,490 occurred in Pernambuco, and 807 were work-related injuries/diseases^19^. This number of occupational accidents and incidents of injury/disease might occur due to non-compliance with health and safety legislation.

In the current study, 75 Pernambuco labor lawsuits were analyzed, and the authors observed that companies complied with RS-17 in only 31% of the analyzed forensic examinations. Failure to comply with the occupational health and safety legislation makes the employer susceptible to the penalties described by the current legislation^20^. Thus, in addition to increasing the risk of accidents and work-related diseases, these companies might suffer financial losses from the fines imposed by the MTE.

In 2010, approximately 2 million labor claims were made in Brazil. The companies were inspected in only 7.5% of these cases. Of these inspections, irregularities were found in 1.7% of all cases. Thus, the inefficiency of the control and supervision agencies appears to encourage non-compliance with the law. In turn, non-compliance triggers labor disputes and burdens the labor courts^6^.

Nevertheless, according to the Superior Labor Court, labor courts imposed fines on the companies that failed to comply with the laws, amounting to R$ 1,228,839,556 and R$ 11,653,029,611.79 in 2009 and 2010, respectively. Therefore, an alarming number of irregularities likely exist with regard to labor law compliance in the working world^6^.

Of the companies that complied with the law, 30% of the forensic examinations analyzed determined a causal nexus. In contrast, organizations that did not comply with RS-17 were shown to have a causal nexus in 71% of the forensic examinations. Based on these results, non-compliance with RS-17 was associated with a 54.8% increase in the probability that a forensic examination would show that an employee's musculoskeletal disorders were directly related to the job. This result corroborated the fact that the experts evaluated the presence and intensity of RSI/WMSD risk factors in an environment where the employee exercised his or her rights.

Importantly, the etiology of RSI/WMSD is multidimensional and, above all, linked to individual, physical and psychosocial factors^21^ as well as being influenced by them. Work-related injuries/diseases are socially produced by organizational determinants of labor and production as well as associated with biomechanical (e.g., physical effort, constrained and static postures, accelerated gestures and repetitive movement) and psychosocial (e.g., work intensity, pressure to meet production targets and cognitive fatigue) risk factors^22^^,^ 23.

Given the above reasons, RS-17 addresses certain aspects related to RSI/WMSD risk factors. This standard establishes the minimum parameters to be adopted in the workplace regarding lifting and transportation as well as the unloading of materials, furniture, and equipment. Furthermore, it recommends the environmental conditions and work organization needed to adapt working conditions to the psychophysiological characteristics of employees to provide comfort and safety as well as to encourage efficient performance. When a company violates this standard, it places the employee's health at risk by fostering the development of occupational injuries/diseases^6^.

A significant correlation was found between RS-17 non-compliance and work absenteeism. This result was expected because employees were exposed to health risk factors when the company did not comply with the standard, thereby increasing the likelihood of developing occupational disease and INSS removal. This issue was important because the INSS granted 319,445 labor accident sickness benefits to employees in 2011, incurring a cost of R$ 297,524,000,000^19^. RSI/WMSDs represent an important public health problem in Brazil^24^ and can lead to a decrease or loss of working capacity^26^; furthermore, these injuries are responsible for the temporary or permanent disability of young adults at a productive age, causing work absenteeism among a large portion of employees^25^ as well as generating costs with severance benefits for the INSS and their employers.

## Conclusions

The results of this study showed that companies' non-compliance with RS-17 significantly increased the likelihood that the forensic examinations conducted by physical therapists would reveal a causal nexus between non-compliance and musculoskeletal disorders. Furthermore, a significant increase in work absenteeism was observed among organizations that did not comply with this standard.

One of the fundamental conclusions of this study concerns the important role that occupational physical therapists play in enforcing employee rights through forensic examination as well as in helping firms to comply with the law via workplace ergonomics evaluations, thereby reducing costs due to fines and lawsuits.

Importantly, this study has limitations, such as the small number of experts and analyzed cases and the fact that it was performed only in Pernambuco, Brazil. However, given the lack of publications on this topic, this study marks the first effort toward a better understanding of this process, which is very important for the Brazilian labor market. Therefore, the authors hope that this study is the beginning of a line of research that seeks a deeper understanding and encourages new studies, especially those with more experts and cases and those conducted in different regions of Brazil for external validation.

## Appendix 1 Expert analysis script.


